# Supplementary material for: Physical Activity in Adults with Schizophrenia and Bipolar Disorder: A Large Cross-Sectional Survey Exploring Patterns, Preferences, Barriers, and Motivating Factors
Source: Int J Environ Res Public Health. 2023 Jan 31;20(3):2548. doi: 10.3390/ijerph20032548 (PMC9916302; doi:10.3390/ijerph20032548)
Supplement: Supplementary file 1 [file ijerph-20-02548-s001.zip › ijerph-2072650-supplementary.pdf]

Supplementary Table S1. Physical activity preferences by gender. Data are reported as % (n).

	Female (n=212)			Male (n=308)			Total (n=529)		
	Agree	Neutral	Disagree	Agree	Neutral	Disagree	Agree	Neutral	Disagree
**I prefer activities that are...**									
Done on my own	54 (114)	24 (51)	17 (36)	66 (202)	20 (61)	12 (36)	60 (318)	22 (118)	14 (72)
Done in a team or group	31 (66)	29 (62)	33 (69)	36 (110)	30 (92)	32 (99)	33 (177)	30 (160)	32 (169)
Done in my own time, at my own pace	75 (159)	13 (27)	6 (13)	74 (227)	17 (51)	7 (22)	74 (391)	15 (81)	7 (35)
Done at a specific time	39 (82)	32 (68)	23 (49)	44 (135)	31 (94)	22 (69)	41 (219)	31 (166)	23 (120)
Done with people around my own age	28 (59)	28 (81)	28 (60)	38 (118)	31 (94)	29 (90)	34 (179)	34 (180)	29 (151)
Done with people of my own gender	25 (53)	38 (81)	30 (64)	23 (72)	41 (127)	32 (98)	24 (129)	40 (212)	31 (162)
Supervised by a qualified trainer	35 (74)	27 (58)	31 (65)	36 (110)	26 (80)	35 (109)	35 (185)	27 (144)	33 (175)
Supervised by a health professional	30 (63)	29 (61)	35 (74)	31 (96)	29 (90)	36 (111)	30 (159)	30 (159)	35 (185)
Done indoors	53 (112)	26 (56)	15 (32)	48 (148)	32 (99)	17 (52)	50 (263)	30 (159)	16 (85)
Done outdoors	50 (106)	29 (62)	16 (34)	58 (178)	24 (75)	16 (48)	54 (287)	27 (142)	16 (82)
Done at home	49 (103)	23 (48)	21 (44)	46 (142)	27 (82)	23 (72)	47 (250)	25 (132)	22 (117)
Done close to home	64 (135)	21 (45)	9 (20)	59 (181)	27 (84)	10 (32)	61 (322)	25 (131)	10 (52)
Low intensity (e.g., walking)	79 (167)	10 (21)	7 (14)	76 (233)	13 (40)	8 (26)	76 (404)	12 (65)	8 (40)
High intensity (e.g., running, aerobics)	27 (58)	19 (41)	48 (102)	29 (89)	19 (57)	49 (152)	28 (149)	19 (101)	49 (257)
Strength based (e.g., weights, strength training)	25 (53)	20 (43)	49 (103)	32 (98)	19 (59)	45 (139)	29 (153)	20 (105)	46 (245)
Sports	19 (41)	21 (44)	51 (108)	36 (110)	17 (52)	43 (131)	29 (152)	19 (101)	46 (241)
**Preferred sources of support:**									
Exercise instructor guiding me through each exercise session	39 (82)	24 (50)	32 (67)	31 (97)	23 (70)	42 (130)	34 (181)	23 (124)	38 (199)
Exercise instructor telling me how to exercise	37 (79)	24 (50)	34 (72)	33 (102)	22 (68)	41 (127)	35 (183)	23 (122)	38 (201)
Support worker coming with me	23 (49)	20 (43)	50 (107)	29 (90)	22 (68)	45 (140)	27 (142)	22 (116)	47 (247)
Psychologist or counsellor telling me to	18 (38)	25 (53)	50 (105)	29 (88)	26 (80)	43 (131)	24 (126)	26 (139)	45 (238)
Social support (e.g., family/friends)[a]	53 (68)	11 (14)	35 (45)	55 (132)	20 (48)	22 (52)	54 (201)	18 (66)	26 (98)
**I prefer doing activity [b]:**									
before 8am	9 (20)			11 (33)			10 (55)		
8-11am	36 (77)			30 (91)			32 (170)		
11am-2pm	42 (90)			38 (118)			40 (209)		
2pm-5pm	33 (69)			35 (109)			34 (182)		
5pm-8pm	21 (45)			20 (63)			21 (111)		
after 8pm	8 (17)			13 (39)			11 (57)		
No preference	12 (26)			22 (68)			19 (98)		

a) asked in second version of survey only, b) can select more than one.

Supplementary Table S2. Physical activity preferences by age. Data are reported as % (n).

	18-34 years (n=81)			35-64 years (n=371)			≥65 years (n=67)		
	Agree	Neutral	Disagree	Agree	Neutral	Disagree	Agree	Neutral	Disagree
**I prefer activities that are...**									
Done on my own	65 (53)	22 (18)	12 (10)	59 (218)	23 (86)	14 (53)	60 (40)	21 (14)	12 (8)
Done in a team or group	36 (29)	32 (26)	32 (26)	35 (129)	30 (111)	32 (120)	22 (15)	31 (21)	33 (22)
Done in my own time, at my own pace	75 (61)	21 (17)	4 (3)	74 (276)	15 (56)	7 (25)	70 (47)	12 (8)	9 (6)
Done at a specific time	48 (39)	32 (26)	20 (16)	43 (160)	31 (114)	23 (84)	25 (17)	34 (23)	27 (18)
Done with people around my own age	37 (30)	40 (32)	23 (19)	31 (116)	36 (132)	30 (110)	39 (26)	22 (15)	33 (22)
Done with people of my own gender	35 (28)	37 (30)	28 (23)	23 (84)	43 (158)	30 (113)	18 (12)	33 (22)	39 (26)
Supervised by a qualified trainer	44 (36)	25 (20)	31 (25)	35 (129)	29 (107)	32 (120)	22 (15)	22 (15)	43 (29)
Supervised by a health professional	28 (23)	35 (28)	37 (30)	32 (119)	30 (113)	33 (123)	18 (12)	25 (17)	46 (31)
Done indoors	52 (42)	33 (27)	15 (12)	51 (191)	30 (113)	15 (54)	37 (25)	27 (18)	28 (19)
Done outdoors	58 (47)	28 (23)	14 (11)	52 (194)	27 (100)	18 (66)	63 (42)	24 (16)	6 (4)
Done at home	48 (39)	28 (23)	23 (19)	48 (177)	24 (89)	23 (84)	39 (26)	30 (20)	21 (14)
Done close to home	63 (51)	28 (23)	9 (7)	61 (227)	24 (90)	11 (40)	57 (38)	25 (17)	7 (5)
Low intensity (e.g., walking)	69 (56)	20 (16)	10 (8)	78 (288)	12 (46)	7 (26)	82 (55)	1 (1)	9 (6)
High intensity (e.g., running, aerobics)	56 (45)	16 (13)	28 (23)	24 (90)	22 (80)	51 (188)	16 (11)	10 (7)	64 (43)
Strength based (e.g., weights, strength training)	51 (41)	22 (18)	26 (21)	27 (102)	21 (78)	48 (177)	10 (7)	10 (7)	67 (45)
Sports	53 (43)	19 (15)	28 (23)	27 (99)	20 (73)	48 (178)	12 (8)	16 (11)	57 (38)
**Preferred sources of support:**									
Exercise instructor guiding me through each exercise session	35 (28)	35 (28)	31 (25)	35 (130)	23 (87)	37 (137)	25 (17)	12 (8)	55 (37)
Exercise instructor telling me how to exercise	40 (32)	32 (26)	28 (23)	34 (127)	23 (87)	38 (141)	27 (18)	12 (8)	55 (37)
Support worker coming with me	27 (22)	27 (22)	46 (37)	28 (105)	21 (79)	46 (170)	18 (12)	19 (13)	57 (38)
Psychologist or counsellor telling me to	27 (22)	22 (18)	51 (41)	25 (94)	28 (103)	43 (159)	12 (8)	21 (14)	55 (37)
Social support (e.g., family/friends)[a]	71 (51)	13 (9)	17 (12)	51 (130)	21 (53)	26 (67)	43 (16)	11 (4)	43 (16)
**I prefer doing activity [b]:**									
before 8am	9 (7)			11 (40)			9 (6)		
8-11am	28 (23)			32 (117)			40 (27)		
11am-2pm	35 (28)			40 (149)			40 (27)		
2pm-5pm	30 (24)			35 (129)			40 (27)		
5pm-8pm	30 (24)			22 (80)			9 (6)		
after 8pm	11 (9)			12 (44)			6 (4)		
No preference	22 (18)			18 (67)			19 (13)		

a) asked in second version of survey only, b) can select more than one.

Supplementary Table S3. Physical activity preferences by physical activity status. Data are reported as % (n).

	Active* (n=304)			Inactive (n=206)		
	**Agree**	**Neutral**	**Disagree**	**Agree**	**Neutral**	**Disagree**
**I prefer activities that are...**						
Done on my own	62 (189)	23 (70)	13 (38)	59 (122)	21 (44)	16 (33)
Done in a team or group	37 (112)	33 (100)	28 (85)	29 (60)	28 (57)	39 (80)
Done in my own time, at my own pace	72 (219)	18 (55)	7 (21)	77 (159)	12 (25)	7 (14)
Done at a specific time	44 (134)	33 (100)	20 (61)	39 (81)	29 (60)	27 (56)
Done with people around my own age	38 (115)	35 (105)	27 (82)	30 (61)	33 (68)	32 (66)
Done with people of my own gender	25 (77)	42 (128)	30 (90)	24 (49)	38 (78)	33 (68)
Supervised by a qualified trainer	32 (98)	32 (96)	34 (102)	39 (81)	22 (46)	33 (69)
Supervised by a health professional	24 (74)	33 (100)	40 (122)	37 (77)	28 (58)	29 (60)
Done indoors	49 (150)	33 (99)	16 (49)	51 (105)	27 (56)	17 (36)
Done outdoors	55 (167)	31 (93)	13 (40)	54 (112)	22 (46)	19 (40)
Done at home	47 (143)	26 (80)	23 (71)	48 (98)	24 (49)	22 (45)
Done close to home	61 (186)	27 (82)	9 (27)	63 (129)	21 (44)	12 (25)
Low intensity (e.g., walking)	76 (232)	13 (40)	8 (25)	79 (162)	10 (21)	7 (14)
High intensity (e.g., running, aerobics)	38 (116)	23 (69)	37 (113)	15 (30)	14 (28)	67 (138)
Strength based (e.g., weights, strength training)	36 (110)	22 (68)	39 (118)	19 (40)	16 (33)	59 (122)
Sports	36 (109)	22 (68)	37 (113)	20 (41)	15 (31)	59 (121)
**Preferred sources of support:**						
Exercise instructor guiding me through each exercise session	32 (97)	26 (78)	40 (121)	38 (79)	21 (43)	36 (74)
Exercise instructor telling me how to exercise	33 (101)	25 (76)	39 (120)	37 (77)	21 (43)	37 (77)
Support worker coming with me	23 (70)	20 (60)	55 (166)	33 (69)	25 (51)	37 (77)
Psychologist or counsellor telling me to	22 (68)	24 (74)	50 (152)	25 (52)	30 (61)	41 (84)
Social support (e.g., family/friends)[a]	55 (118)	17 (36)	26 (56)	53 (78)	18 (27)	28 (41)
**I prefer doing activity [b]:**						
before 8am	13 (38)			7 (14)		
8-11am	34 (104)			29 (59)		
11am-2pm	41 (125)			37 (76)		
2pm-5pm	34 (104)			34 (70)		
5pm-8pm	22 (68)			18 (38)		
after 8pm	11 (34)			10 (20)		
No preference	15 (47)			22 (45)		

*Active is defined as ≥150 min/week of aerobic physical activity of at least a moderate intensity (excluding walking). a) asked in second version of survey only, b) can select more than one.

Supplementary Table S4. Physical activity preferences by mental health status. Data are reported as % (n).

	**Excellent-good (n=199)**			**Moderate (n=166)**			**Poor-very poor (n=157)**		
	**Agree**	**Neutral**	**Disagree**	**Agree**	**Neutral**	**Disagree**	**Agree**	**Neutral**	**Disagree**
**I prefer activities that are...**									
Done on my own	56 (111)	24 (48)	17 (34)	66 (109)	19 (31)	10 (17)	60 (94)	24 (38)	13 (21)
Done in a team or group	45 (89)	27 (54)	25 (49)	34 (57)	30 (49)	31 (52)	20 (31)	36 (56)	41 (64)
Done in my own time, at my own pace	70 (140)	18 (35)	8 (16)	76 (126)	12 (20)	6 (10)	76 (119)	17 (26)	6 (9)
Done at a specific time	40 (80)	28 (56)	28 (55)	45 (74)	30 (49)	20 (34)	39 (62)	38 (59)	19 (30)
Done with people around my own age	41 (82)	31 (61)	26 (51)	36 (59)	30 (50)	30 (50)	24 (37)	43 (67)	31 (48)
Done with people of my own gender	24 (48)	41 (82)	31 (61)	24 (40)	41 (68)	29 (48)	25 (39)	38 (60)	33 (52)
Supervised by a qualified trainer	41 (82)	26 (51)	29 (58)	31 (52)	27 (45)	37 (61)	31 (48)	29 (46)	35 (55)
Supervised by a health professional	32 (64)	27 (54)	36 (71)	31 (52)	29 (48)	36 (59)	27 (42)	34 (54)	34 (54)
Done indoors	49 (97)	32 (64)	16 (31)	54 (90)	25 (42)	16 (27)	46 (72)	34 (53)	16 (25)
Done outdoors	56 (112)	28 (55)	14 (27)	60 (99)	20 (34)	17 (28)	45 (71)	34 (53)	17 (27)
Done at home	47 (93)	26 (52)	23 (46)	51 (84)	24 (40)	19 (31)	45 (71)	25 (40)	25 (39)
Done close to home	58 (116)	26 (51)	12 (24)	67 (112)	19 (32)	8 (13)	57 (90)	30 (47)	9 (14)
Low intensity (e.g., walking)	78 (155)	12 (24)	9 (18)	78 (130)	10 (17)	5 (8)	74 (116)	15 (23)	8 (12)
High intensity (e.g., running, aerobics)	34 (67)	21 (42)	43 (85)	28 (46)	16 (27)	51 (84)	23 (36)	19 (30)	54 (85)
Strength based (e.g., weights, strength training)	36 (72)	18 (35)	43 (85)	27 (45)	19 (32)	48 (79)	22 (35)	23 (36)	50 (79)
Sports	39 (78)	18 (35)	36 (72)	27 (44)	17 (28)	50 (83)	18 (28)	24 (38)	53 (83)
**Preferred sources of support:**									
Exercise instructor guiding me through each exercise session	40 (80)	21 (41)	36 (71)	31 (51)	25 (41)	39 (65)	31 (48)	26 (41)	38 (60)
Exercise instructor telling me how to exercise	42 (83)	20 (39)	35 (70)	34 (57)	25 (41)	37 (62)	27 (43)	25 (40)	42 (66)
Support worker coming with me	27 (54)	17 (33)	53 (105)	27 (44)	23 (39)	44 (73)	28 (44)	27 (43)	41 (65)
Psychologist or counsellor telling me to	21 (42)	23 (46)	52 (103)	23 (39)	28 (46)	43 (71)	29 (45)	29 (45)	39 (61)
Social support (e.g., family/friends)[a]	59 (84)	13 (18)	28 (40)	50 (58)	25 (29)	22 (25)	52 (59)	15 (17)	29 (33)
**I prefer doing activity [b]:**									
before 8am	13 (26)			10 (17)			8 (12)		
8-11am	29 (58)			37 (61)			31 (49)		
11am-2pm	41 (81)			39 (65)			37 (58)		
2pm-5pm	33 (65)			36 (60)			34 (53)		
5pm-8pm	20 (40)			18 (30)			25 (40)		
after 8pm	13 (26)			8 (14)			11 (17)		
No preference	22 (43)			17 (29)			17 (26)		

a) asked in second version of survey only, b) can select more than one.

Supplementary Table S5. Physical activity barriers by gender. Data are reported as % (n).

	Female (n=212)			Male (n=308)			Total (n=529)		
**Barrier**	**Agree**	**Neutral**	**Disagree**	**Agree**	**Neutral**	**Disagree**	**Agree**	**Neutral**	**Disagree**
Poor physical health	53 (112)	12 (25)	31 (66)	48 (148)	18 (54)	32 (100)	50 (266)	15 (79)	32 (168)
Lack of money	40 (84)	17 (36)	39 (83)	30 (93)	20 (62)	46 (143)	34 (180)	19 (101)	43 (228)
Poor mental health	56 (119)	15 (31)	25 (52)	53 (162)	16 (49)	30 (91)	54 (285)	16 (83)	27 (144)
I can't get motivated	58 (123)	17 (36)	20 (43)	53 (164)	18 (54)	27 (82)	55 (290)	18 (94)	24 (126)
I don't like the gym	53 (112)	17 (36)	25 (53)	44 (136)	20 (62)	34 (104)	48 (252)	19 (100)	30 (159)
Problems with transport	34 (72)	17 (35)	44 (93)	27 (84)	16 (50)	54 (166)	30 (159)	17 (88)	49 (261)
I'm too shy or embarrassed	35 (75)	17 (35)	43 (91)	27 (82)	24 (74)	46 (143)	31 (162)	21 (110)	45 (236)
I can't get organised	25 (54)	21 (45)	47 (100)	29 (90)	26 (81)	42 (128)	28 (146)	25 (130)	43 (230)
My weight	45 (96)	15 (32)	34 (73)	30 (91)	17 (52)	50 (153)	36 (189)	16 (87)	43 (229)
I'm too tired	55 (116)	18 (38)	23 (49)	48 (147)	20 (62)	30 (91)	50 (266)	19 (103)	27 (142)
Medication side-effects	43 (91)	15 (32)	38 (80)	44 (136)	19 (59)	34 (105)	43 (230)	18 (94)	35 (187)
I have an injury	28 (60)	8 (17)	58 (124)	31 (95)	14 (44)	52 (159)	30 (159)	12 (62)	54 (286)
I don't have the right clothes/ shoes	18 (39)	17 (35)	60 (127)	17 (51)	17 (53)	62 (192)	18 (94)	17 (90)	61 (321)
I'm not sure what to do	25 (53)	20 (42)	50 (106)	23 (72)	20 (62)	53 (162)	24 (128)	20 (106)	51 (271)
Facilities are too far away	30 (64)	18 (38)	46 (97)	22 (69)	18 (54)	57 (175)	26 (136)	18 (94)	52 (275)
Exercise makes me exhausted	37 (78)	19 (40)	39 (83)	38 (117)	22 (67)	37 (115)	37 (198)	20 (108)	38 (202)
Lack of skill	24 (50)	23 (49)	47 (99)	22 (69)	21 (65)	53 (164)	23 (122)	22 (115)	50 (267)
My age	20 (43)	16 (34)	59 (125)	23 (70)	19 (57)	55 (170)	22 (115)	18 (93)	57 (299)
Social demands	18 (39)	15 (31)	60 (128)	15 (46)	23 (72)	58 (178)	16 (86)	20 (106)	59 (310)
I'm afraid of getting injured	17 (37)	13 (27)	63 (133)	22 (67)	17 (51)	58 (179)	20 (106)	15 (80)	60 (316)
Lack of time	23 (48)	16 (34)	56 (118)	18 (55)	17 (52)	61 (189)	20 (104)	17 (90)	59 (310)
I do not enjoy physical activity	26 (56)	16 (34)	52 (110)	25 (76)	18 (54)	54 (166)	26 (135)	17 (90)	53 (279)
The mental health service takes up too much of my time	8 (18)	9 (20)	76 (162)	15 (46)	14 (44)	67 (206)	12 (64)	12 (66)	71 (374)
Work demands	15 (31)	10 (22)	68 (145)	12 (37)	16 (49)	68 (210)	13 (70)	14 (73)	68 (359)
Lack of access to childcare	8 (16)	8 (16)	78 (166)	6 (19)	12 (36)	77 (237)	7 (35)	11 (56)	77 (407)
**Asked in second version of survey only:**									
I don't have anyone to do physical activity with	24 (31)	13 (17)	46 (59)	25 (59)	12 (28)	50 (118)	25 (93)	12 (46)	48 (178)
The thought of physical activity makes me worry	22 (28)	16 (20)	46 (59)	20 (48)	12 (28)	54 (128)	21 (78)	13 (49)	51 (189)
I don't think that physical activity will benefit me	9 (11)	13 (17)	62 (80)	11 (26)	8 (20)	66 (158)	10 (37)	10 (38)	65 (242)
My close contacts don't support or encourage my physical activity	9 (12)	13 (17)	60 (77)	11 (27)	18 (44)	56 (134)	10 (39)	17 (62)	57 (215)
I get easily distracted from the physical activity I have planned	29 (37)	15 (19)	39 (50)	24 (57)	17 (41)	45 (107)	25 (95)	16 (61)	43 (160)

I don't make plans for doing physical activity	33 (42)	12 (16)	37 (48)	34 (82)	14 (34)	37 (87)	33 (125)	14 (53)	36 (136)
I don't think physical activity is important	8 (10)	9 (11)	66 (85)	13 (31)	8 (20)	64 (152)	11 (41)	9 (32)	64 (241)
Physical activity is not something I do automatically	39 (50)	12 (16)	32 (41)	34 (82)	16 (37)	35 (84)	36 (133)	14 (54)	34 (128)
Fear of judgement/stigma because of mental (or physical) health problems	26 (34)	11 (14)	45 (58)	29 (69)	13 (32)	43 (102)	28 (104)	13 (47)	44 (163)

Supplementary Table S6. Physical activity barriers by age. Data are reported as % (n).

	**18-34 years (n=81)**			**35-64 years (n=371)**			**≥65 years (n=67)**		
	**Agree**	**Neutral**	**Disagree**	**Agree**	**Neutral**	**Disagree**	**Agree**	**Neutral**	**Disagree**
Poor physical health	44 (36)	22 (18)	32 (26)	51 (191)	15 (55)	31 (116)	49 (33)	9 (6)	37 (25)
Lack of money	36 (29)	26 (21)	37 (30)	36 (135)	19 (69)	42 (156)	18 (12)	13 (9)	61 (41)
Poor mental health	52 (42)	22 (18)	25 (20)	57 (212)	15 (56)	26 (95)	37 (25)	13 (9)	40 (27)
I can't get motivated	62 (50)	23 (19)	15 (12)	57 (211)	17 (62)	23 (87)	37 (25)	18 (12)	37 (25)
I don't like the gym	40 (32)	27 (22)	32 (26)	49 (183)	18 (66)	30 (113)	46 (31)	18 (12)	28 (19)
Problems with transport	25 (20)	15 (12)	59 (48)	32 (120)	17 (62)	48 (178)	21 (14)	19 (13)	51 (34)
I'm too shy or embarrassed	32 (26)	25 (20)	42 (34)	32 (118)	22 (80)	44 (162)	21 (14)	12 (8)	58 (39)
I can't get organised	27 (22)	25 (20)	47 (38)	29 (108)	26 (96)	42 (155)	19 (13)	18 (12)	52 (35)
My weight	26 (21)	19 (15)	54 (44)	39 (143)	17 (62)	41 (153)	31 (21)	13 (9)	46 (31)
I'm too tired	51 (41)	20 (16)	28 (23)	52 (193)	20 (76)	25 (91)	42 (28)	15 (10)	39 (26)
Medication side-effects	41 (33)	28 (23)	30 (24)	47 (175)	15 (57)	35 (131)	28 (19)	18 (12)	46 (31)
I have an injury	27 (22)	12 (10)	59 (48)	30 (113)	12 (46)	54 (201)	33 (22)	9 (6)	49 (33)
I don't have the right clothes/ shoes	15 (12)	19 (15)	65 (53)	19 (72)	16 (61)	61 (226)	12 (8)	19 (13)	60 (40)
I'm not sure what to do	22 (18)	17 (14)	59 (48)	26 (98)	20 (76)	50 (185)	15 (10)	21 (14)	55 (37)
Facilities are too far away	21 (17)	16 (13)	62 (50)	27 (99)	18 (66)	52 (194)	24 (16)	19 (13)	46 (31)
Exercise makes me exhausted	32 (26)	19 (15)	48 (39)	39 (144)	22 (82)	36 (134)	37 (25)	15 (10)	40 (27)
Lack of skill	23 (19)	19 (15)	58 (47)	25 (91)	22 (80)	50 (185)	12 (8)	28 (19)	51 (34)
My age	2 (2)	11 (9)	85 (69)	25 (91)	20 (73)	53 (196)	28 (19)	16 (11)	46 (31)
Social demands	17 (14)	19 (15)	63 (51)	18 (65)	20 (73)	58 (217)	7 (5)	25 (17)	58 (39)
I'm afraid of getting injured	12 (10)	19 (15)	68 (55)	22 (81)	15 (55)	60 (221)	18 (12)	15 (10)	55 (37)
Lack of time	31 (25)	15 (12)	54 (44)	18 (68)	17 (64)	60 (224)	13 (9)	19 (13)	58 (39)
I do not enjoy physical activity	19 (15)	17 (14)	62 (50)	27 (100)	17 (64)	52 (194)	25 (17)	16 (11)	49 (33)
The mental health service takes up too much of my time	10 (8)	14 (11)	75 (61)	14 (52)	12 (44)	70 (261)	3 (2)	15 (10)	73 (49)
Work demands	19 (15)	14 (11)	67 (54)	13 (48)	15 (55)	68 (253)	6 (4)	9 (6)	75 (50)
Lack of access to childcare	9 (7)	20 (16)	70 (57)	6 (24)	9 (34)	79 (294)	3 (2)	7 (5)	79 (53)
**Asked in second version of survey only:**									
I don't have anyone to do physical activity with	28 (20)	10 (7)	47 (34)	24 (61)	13 (33)	48 (124)	24 (9)	16 (6)	51 (19)
The thought of physical activity makes me worry	18 (13)	8 (6)	58 (42)	22 (57)	15 (39)	47 (121)	19 (7)	8 (3)	65 (24)
I don't think that physical activity will benefit me	8 (6)	10 (7)	67 (48)	11 (29)	10 (25)	64 (164)	3 (1)	14 (5)	76 (28)
My close contacts don't support or encourage my physical activity	14 (10)	17 (12)	54 (39)	11 (27)	18 (46)	57 (145)	5 (2)	8 (3)	76 (28)
I get easily distracted from the physical activity I have planned	31 (22)	15 (11)	39 (28)	25 (64)	17 (43)	43 (110)	22 (8)	16 (6)	51 (19)

I don't make plans for doing physical activity	32 (23)	13 (9)	39 (28)	35 (90)	15 (38)	35 (89)	27 (10)	16 (6)	49 (18)
I don't think physical activity is important	8 (6)	7 (5)	68 (49)	13 (32)	9 (23)	63 (162)	5 (2)	11 (4)	73 (27)
Physical activity is not something I do automatically	32 (23)	15 (11)	36 (26)	38 (96)	14 (35)	34 (87)	32 (12)	19 (7)	38 (14)
Fear of judgement/stigma because of mental (or physical) health problems	26 (19)	14 (10)	43 (31)	30 (77)	12 (30)	43 (110)	19 (7)	11 (4)	59 (22)

Supplementary Table S7. Physical activity barriers by physical activity status. Data are reported as % (n).

	**Active* (n=304)**			**Inactive (n=206)**		
	**Agree**	**Neutral**	**Disagree**	**Agree**	**Neutral**	**Disagree**
Poor physical health	41 (124)	16 (49)	41 (124)	65 (133)	13 (26)	20 (41)
Lack of money	33 (100)	19 (57)	46 (139)	35 (72)	21 (43)	40 (83)
Poor mental health	51 (156)	18 (55)	29 (88)	59 (121)	12 (25)	24 (50)
I can't get motivated	48 (145)	20 (60)	30 (90)	67 (138)	15 (30)	16 (32)
I don't like the gym	41 (126)	21 (64)	35 (106)	56 (116)	17 (34)	23 (48)
Problems with transport	28 (84)	16 (50)	53 (160)	33 (68)	16 (33)	47 (96)
I'm too shy or embarrassed	29 (87)	22 (67)	46 (141)	36 (74)	18 (38)	41 (85)
I can't get organised	24 (74)	23 (70)	50 (151)	33 (69)	28 (57)	34 (70)
My weight	29 (88)	16 (50)	51 (154)	46 (95)	16 (32)	34 (70)
I'm too tired	45 (138)	20 (61)	32 (96)	58 (119)	19 (40)	20 (41)
Medication side-effects	39 (119)	20 (60)	38 (117)	50 (102)	16 (32)	32 (65)
I have an injury	32 (98)	14 (42)	51 (155)	26 (53)	9 (18)	61 (125)
I don't have the right clothes/ shoes	16 (49)	17 (52)	63 (193)	19 (39)	17 (35)	59 (121)
I'm not sure what to do	20 (60)	19 (59)	57 (174)	31 (64)	21 (43)	43 (88)
Facilities are too far away	24 (72)	17 (52)	55 (168)	30 (61)	18 (37)	48 (99)
Exercise makes me exhausted	27 (83)	23 (69)	47 (143)	52 (107)	17 (36)	26 (54)
Lack of skill	21 (63)	22 (66)	54 (163)	27 (56)	22 (46)	46 (94)
My age	19 (59)	18 (56)	59 (178)	26 (53)	17 (35)	53 (110)
Social demands	16 (48)	20 (60)	61 (185)	18 (37)	20 (41)	57 (117)
I'm afraid of getting injured	16 (49)	17 (53)	62 (189)	26 (53)	13 (26)	56 (116)
Lack of time	18 (56)	18 (56)	60 (181)	23 (47)	15 (30)	58 (119)
I do not enjoy physical activity	16 (50)	16 (48)	63 (193)	39 (81)	19 (40)	37 (76)
The mental health service takes up too much of my time	11 (33)	14 (44)	71 (215)	14 (28)	11 (22)	71 (146)
Work demands	15 (46)	15 (45)	66 (200)	11 (23)	12 (24)	72 (149)
Lack of access to childcare	7 (20)	12 (35)	77 (234)	7 (14)	9 (18)	79 (162)
**Asked in second version of survey only:**						
I don't have anyone to do physical activity with	20 (42)	13 (27)	53 (114)	32 (47)	13 (19)	40 (59)
The thought of physical activity makes me worry	15 (33)	14 (29)	56 (120)	30 (44)	13 (19)	42 (62)
I don't think that physical activity will benefit me	7 (15)	8 (18)	70 (149)	15 (22)	13 (19)	57 (85)
My close contacts don't support or encourage my physical activity	8 (18)	16 (34)	60 (129)	14 (21)	18 (26)	53 (79)
I get easily distracted from the physical activity I have planned	21 (46)	17 (36)	46 (98)	31 (46)	16 (24)	39 (57)

I don't make plans for doing physical activity	18 (38)	18 (39)	48 (102)	55 (81)	9 (13)	22 (32)
I don't think physical activity is important	9 (20)	5 (10)	70 (149)	12 (18)	15 (22)	58 (86)
Physical activity is not something I do automatically	25 (54)	17 (36)	42 (90)	50 (74)	11 (17)	24 (35)
Fear of judgement/stigma because of mental (or physical) health problems	25 (53)	14 (31)	45 (96)	34 (50)	10 (15)	41 (60)

*Active is defined as ≥150 min/week of aerobic physical activity of at least a moderate intensity (excluding walking).

Supplementary Table S8. Physical activity barriers by mental health status. Data are reported as % (n).

	Excellent-good (n=199)			Moderate (n=166)			Poor-very poor (n=157)		
	Agree	Neutral	Disagree	Agree	Neutral	Disagree	Agree	Neutral	Disagree
Poor physical health	37 (74)	14 (27)	46 (92)	55 (91)	17 (29)	24 (40)	63 (99)	13 (21)	22 (34)
Lack of money	28 (56)	17 (33)	53 (105)	37 (61)	20 (33)	38 (63)	39 (61)	22 (34)	36 (57)
Poor mental health	29 (58)	17 (33)	51 (101)	59 (98)	20 (34)	17 (29)	80 (125)	10 (16)	8 (13)
I can't get motivated	40 (80)	15 (29)	43 (85)	57 (94)	22 (36)	17 (28)	71 (111)	18 (29)	8 (12)
I don't like the gym	38 (76)	17 (34)	43 (85)	49 (82)	22 (36)	25 (41)	59 (92)	18 (29)	20 (31)
Problems with transport	24 (47)	10 (20)	63 (126)	30 (50)	20 (34)	45 (74)	39 (62)	20 (32)	37 (58)
I'm too shy or embarrassed	19 (37)	19 (38)	59 (118)	31 (52)	24 (40)	40 (66)	46 (73)	19 (30)	31 (49)
I can't get organised	19 (37)	19 (37)	60 (119)	31 (51)	30 (50)	34 (56)	36 (56)	27 (42)	34 (53)
My weight	25 (50)	16 (32)	55 (109)	37 (62)	17 (29)	39 (65)	48 (75)	16 (25)	34 (53)
I'm too tired	36 (72)	21 (42)	41 (81)	50 (83)	24 (40)	21 (35)	69 (108)	12 (19)	16 (25)
Medication side-effects	31 (62)	15 (30)	52 (103)	39 (65)	25 (41)	31 (51)	64 (100)	15 (23)	20 (31)
I have an injury	27 (54)	8 (15)	63 (125)	30 (50)	13 (22)	51 (84)	34 (54)	15 (24)	47 (74)
I don't have the right clothes/ shoes	16 (31)	10 (19)	72 (143)	14 (23)	23 (38)	57 (94)	25 (40)	20 (32)	51 (80)
I'm not sure what to do	20 (40)	15 (30)	62 (124)	22 (36)	23 (39)	48 (79)	33 (52)	22 (35)	41 (65)
Facilities are too far away	23 (46)	11 (22)	63 (125)	21 (35)	25 (42)	47 (78)	34 (54)	19 (30)	43 (68)
Exercise makes me exhausted	28 (56)	15 (29)	54 (108)	39 (64)	27 (45)	29 (48)	49 (77)	20 (32)	28 (44)
Lack of skill	17 (33)	17 (34)	63 (125)	23 (39)	24 (40)	46 (76)	31 (49)	25 (40)	40 (63)
My age	18 (36)	9 (18)	70 (139)	20 (34)	24 (40)	49 (82)	28 (44)	22 (34)	47 (74)
Social demands	13 (26)	14 (28)	69 (138)	14 (24)	25 (42)	54 (89)	22 (35)	22 (35)	51 (80)
I'm afraid of getting injured	18 (36)	11 (21)	68 (135)	17 (29)	9 (32)	55 (92)	26 (41)	17 (26)	54 (85)
Lack of time	19 (37)	13 (25)	66 (132)	22 (37)	19 (32)	52 (86)	19 (30)	20 (32)	56 (88)
I do not enjoy physical activity	18 (35)	13 (25)	66 (132)	25 (42)	22 (36)	47 (78)	35 (55)	18 (29)	42 (66)
The mental health service takes up too much of my time	13 (26)	10 (19)	74 (148)	8 (14)	14 (23)	70 (117)	15 (24)	15 (23)	67 (105)
Work demands	13 (25)	15 (29)	70 (139)	15 (25)	16 (27)	61 (101)	13 (20)	10 (16)	73 (115)
Lack of access to childcare	6 (11)	11 (22)	79 (158)	5 (9)	10 (17)	75 (125)	10 (15)	10 (16)	76 (120)
**Asked in second version of survey only:**									
I don't have anyone to do physical activity with	21 (30)	8 (12)	56 (79)	25 (29)	16 (18)	43 (50)	30 (34)	13 (15)	42 (48)
The thought of physical activity makes me worry	11 (15)	8 (11)	67 (95)	17 (20)	20 (23)	46 (53)	38 (43)	12 (14)	35 (40)
I don't think that physical activity will benefit me	10 (14)	4 (6)	71 (101)	9 (10)	14 (16)	62 (71)	11 (13)	14 (16)	60 (68)
My close contacts don't support or encourage my physical activity	8 (12)	11 (16)	65 (93)	11 (13)	18 (21)	55 (63)	12 (14)	22 (25)	50 (57)
I get easily distracted from the physical activity I have planned	16 (23)	15 (21)	54 (77)	30 (34)	20 (23)	34 (39)	32 (37)	15 (17)	38 (43)

I don't make plans for doing physical activity	23 (32)	13 (19)	49 (69)	37 (42)	15 (17)	31 (36)	44 (50)	14 (16)	27 (31)
I don't think physical activity is important	11 (15)	5 (7)	70 (99)	11 (13)	10 (11)	62 (71)	11 (12)	12 (14)	61 (70)
Physical activity is not something I do automatically	28 (40)	10 (14)	47 (67)	35 (40)	19 (22)	30 (34)	46 (52)	16 (18)	23 (26)
Fear of judgement/stigma because of mental (or physical) health problems	17 (24)	8 (11)	61 (86)	24 (28)	16 (18)	43 (50)	46 (52)	15 (17)	23 (26)

Supplementary Table S9. Motivating factors by gender. Data are reported as % (n).

	**Female (n=212)**			**Male (n=308)**			**Total (n=529)**		
	**Agree**	**Neutral**	**Disagree**	**Agree**	**Neutral**	**Disagree**	**Agree**	**Neutral**	**Disagree**
To improve my mental health	84 (179)	7 (15)	5 (10)	83 (257)	8 (26)	6 (18)	84 (443)	8 (42)	5 (28)
To get stronger and fitter	81 (171)	12 (26)	4 (9)	82 (252)	11 (35)	4 (13)	81 (431)	12 (61)	4 (22)
To get out of the house	79 (168)	8 (17)	8 (18)	81 (250)	11 (33)	6 (20)	79 (420)	10 (55)	7 (39)
To have more energy	78 (165)	12 (26)	5 (11)	77 (238)	12 (37)	7 (23)	77 (408)	12 (66)	6 (34)
To cope with life stresses better	76 (161)	10 (22)	8 (17)	75 (230)	13 (39)	9 (29)	74 (394)	12 (66)	9 (46)
To improve my posture	60 (127)	19 (40)	16 (34)	64 (196)	18 (55)	15 (45)	62 (328)	19 (98)	15 (79)
To relax	68 (145)	15 (32)	12 (25)	72 (221)	13 (41)	11 (35)	70 (369)	14 (74)	12 (64)
To balance other things I do (e.g., smoking, overeating)	52 (110)	15 (31)	28 (59)	52 (159)	18 (55)	27 (83)	52 (274)	17 (88)	27 (143)
To prevent sickness and illness	58 (123)	21 (45)	16 (34)	70 (215)	16 (49)	11 (35)	65 (344)	18 (96)	13 (69)
To lose weight	73 (155)	9 (20)	13 (27)	68 (210)	12 (36)	18 (54)	71 (373)	11 (56)	15 (81)
To improve how I look	62 (132)	15 (32)	18 (39)	64 (198)	16 (48)	17 (52)	64 (336)	16 (82)	17 (91)
To help me sleep	62 (131)	15 (32)	17 (35)	68 (208)	14 (44)	15 (46)	65 (342)	15 (79)	16 (83)
To make new friends	48 (101)	21 (45)	26 (56)	52 (160)	23 (72)	22 (67)	50 (264)	23 (120)	24 (125)
To recover from illness/injury	41 (87)	26 (55)	28 (59)	49 (152)	20 (62)	27 (84)	46 (243)	22 (119)	27 (145)
To spend time with others (family, friends, partner)	53 (113)	17 (36)	24 (51)	56 (171)	21 (65)	20 (61)	54 (285)	20 (104)	22 (116)
To counteract the side-effects of medications	42 (89)	24 (51)	29 (61)	47 (146)	22 (68)	27 (82)	45 (239)	23 (121)	27 (145)

Supplementary Table S10. Motivating factors by age. Data are reported as % (n).

	18-34 years (n=81)			35-64 years (n=371)			≥65 years (n=67)		
	**Agree**	**Neutral**	**Disagree**	**Agree**	**Neutral**	**Disagree**	**Agree**	**Neutral**	**Disagree**
To improve my mental health	88 (71)	6 (5)	5 (4)	84 (313)	9 (34)	5 (17)	76 (51)	4 (3)	10 (7)
To get stronger and fitter	88 (71)	9 (7)	2 (2)	81 (300)	13 (47)	4 (16)	78 (52)	10 (7)	6 (4)
To get out of the house	79 (64)	9 (7)	12 (10)	80 (295)	12 (44)	6 (23)	81 (54)	4 (3)	9 (6)
To have more energy	86 (70)	5 (4)	7 (6)	77 (287)	13 (50)	6 (24)	67 (45)	16 (11)	6 (4)
To cope with life stresses better	79 (64)	11 (9)	9 (7)	76 (281)	12 (46)	9 (33)	66 (44)	15 (10)	9 (6)
To improve my posture	65 (53)	14 (11)	20 (16)	63 (232)	20 (74)	14 (53)	57 (38)	18 (12)	15 (10)
To relax	67 (54)	15 (12)	16 (13)	72 (268)	14 (53)	11 (40)	63 (42)	12 (8)	15 (10)
To balance other things I do (e.g., smoking, overeating)	58 (47)	17 (14)	23 (19)	54 (199)	16 (61)	27 (99)	33 (22)	18 (12)	37 (25)
To prevent sickness and illness	65 (53)	19 (15)	15 (12)	65 (241)	18 (68)	14 (53)	66 (44)	18 (12)	6 (4)
To lose weight	81 (66)	5 (4)	14 (11)	71 (264)	12 (46)	14 (53)	55 (37)	7 (5)	25 (17)
To improve how I look	81 (66)	5 (4)	12 (10)	63 (233)	17 (63)	18 (65)	45 (30)	22 (15)	24 (16)
To help me sleep	78 (63)	9 (7)	12 (10)	64 (237)	16 (61)	17 (62)	55 (37)	13 (9)	16 (11)
To make new friends	53 (43)	19 (15)	28 (23)	49 (181)	25 (92)	23 (87)	55 (37)	16 (11)	19 (13)
To recover from illness/injury	58 (47)	11 (9)	30 (24)	44 (165)	24 (90)	28 (105)	43 (29)	24 (16)	22 (15)
To spend time with others (family, friends, partner)	60 (49)	19 (15)	20 (16)	53 (197)	22 (80)	22 (82)	54 (36)	9 (6)	25 (17)
To counteract the side-effects of medications	52 (42)	17 (14)	28 (23)	45 (168)	25 (91)	27 (101)	37 (25)	21 (14)	30 (20)

Supplementary Table S11. Motivating factors by physical activity status. Data are reported as % (n).

	Active* (n=273)			Inactive (n=229)		
	**Agree**	**Neutral**	**Disagree**	**Agree**	**Neutral**	**Disagree**
To improve my mental health	88 (241)	6 (17)	4 (11)	79 (180)	10 (24)	7 (17)
To get stronger and fitter	86 (236)	10 (27)	2 (6)	77 (176)	14 (31)	7 (16)
To get out of the house	83 (227)	9 (24)	6 (17)	76 (175)	13 (29)	8 (19)
To have more energy	81 (220)	12 (33)	5 (14)	73 (167)	14 (33)	9 (20)
To cope with life stresses better	81 (221)	10 (27)	7 (19)	67 (154)	17 (39)	11 (26)
To improve my posture	64 (176)	20 (55)	13 (35)	61 (139)	16 (37)	18 (42)
To relax	73 (200)	13 (36)	11 (31)	66 (152)	15 (34)	14 (32)
To balance other things I do (e.g., smoking, overeating)	55 (151)	15 (40)	28 (76)	47 (108)	20 (46)	28 (63)
To prevent sickness and illness	69 (188)	18 (50)	11 (29)	62 (143)	18 (42)	15 (35)
To lose weight	72 (196)	12 (34)	14 (37)	71 (163)	8 (19)	17 (39)
To improve how I look	64 (176)	19 (52)	14 (39)	65 (148)	13 (29)	19 (44)
To help me sleep	70 (190)	15 (40)	12 (34)	59 (134)	17 (39)	21 (47)
To make new friends	55 (150)	23 (63)	20 (55)	44 (101)	22 (50)	30 (68)
To recover from illness/injury	48 (130)	23 (62)	27 (74)	44 (100)	22 (51)	30 (69)
To spend time with others (family, friends, partner)	57 (155)	20 (54)	21 (57)	50 (114)	21 (47)	25 (57)
To counteract the side-effects of medications	45 (123)	25 (69)	27 (73)	44 (101)	21 (47)	31 (71)

*Active is defined as ≥150 min/week of aerobic physical activity of at least a moderate intensity (excluding walking).

Supplementary Table S12. Motivating factors by mental health status. Data are reported as % (n).

	**Excellent-Good (n=199)**			**Moderate (n=166)**			**Poor-very Poor (n=157)**		
	**Agree**	**Neutral**	**Disagree**	**Agree**	**Neutral**	**Disagree**	**Agree**	**Neutral**	**Disagree**
To improve my mental health	82 (164)	8 (16)	7 (13)	84 (140)	7 (12)	4 (7)	85 (133)	9 (14)	5 (8)
To get stronger and fitter	84 (168)	9 (18)	4 (8)	78 (130)	14 (23)	5 (8)	82 (128)	12 (19)	4 (6)
To get out of the house	85 (169)	8 (16)	6 (11)	80 (132)	9 (15)	7 (12)	73 (115)	15 (24)	10 (15)
To have more energy	78 (155)	11 (21)	8 (16)	77 (128)	13 (22)	5 (8)	77 (121)	14 (22)	6 (10)
To cope with life stresses better	79 (157)	8 (15)	10 (20)	75 (124)	14 (23)	7 (11)	69 (109)	17 (27)	10 (15)
To improve my posture	68 (135)	13 (25)	16 (32)	59 (98)	20 (34)	14 (23)	58 (91)	24 (38)	15 (23)
To relax	77 (153)	9 (18)	12 (23)	69 (115)	15 (25)	9 (15)	61 (96)	20 (31)	17 (26)
To balance other things I do (e.g., smoking, overeating)	53 (105)	14 (28)	30 (59)	50 (83)	19 (32)	25 (41)	54 (84)	17 (27)	26 (41)
To prevent sickness and illness	71 (142)	13 (26)	13 (25)	63 (105)	20 (34)	11 (19)	60 (94)	22 (34)	16 (25)
To lose weight	75 (150)	8 (15)	15 (30)	69 (115)	11 (18)	16 (26)	67 (105)	14 (22)	15 (24)
To improve how I look	70 (140)	11 (21)	16 (32)	58 (97)	17 (29)	19 (31)	61 (95)	19 (30)	18 (28)
To help me sleep	69 (137)	14 (27)	14 (27)	61 (102)	14 (24)	18 (30)	64 (100)	17 (27)	16 (25)
To make new friends	61 (122)	19 (37)	17 (34)	46 (76)	24 (40)	26 (43)	41 (64)	26 (41)	30 (47)
To recover from illness/injury	49 (98)	22 (43)	26 (52)	43 (71)	25 (42)	25 (42)	45 (70)	21 (33)	32 (50)
To spend time with others (family, friends, partner)	62 (123)	19 (37)	16 (32)	49 (82)	19 (32)	25 (41)	49 (77)	22 (35)	26 (41)
To counteract the side-effects of medications	47 (94)	20 (39)	30 (59)	43 (71)	24 (40)	27 (45)	46 (72)	25 (40)	25 (40)
